# Intelligent on-demand design of phononic metamaterials

**DOI:** 10.1515/nanoph-2021-0639

**Published:** 2022-01-04

**Authors:** Yabin Jin, Liangshu He, Zhihui Wen, Bohayra Mortazavi, Hongwei Guo, Daniel Torrent, Bahram Djafari-Rouhani, Timon Rabczuk, Xiaoying Zhuang, Yan Li

**Affiliations:** School of Aerospace Engineering and Applied Mechanics, Tongji University, 200092, Shanghai, China; Department of Mathematics and Physics, Institute of Photonics, Leibniz University Hannover, Hannover, Germany; GROC-UJI, Institut de Noves Tecnologies de la Imatge, Universitat Jaume I, 12080, Castello, Spain; Département de Physique, Institut d’Electronique, de Microélectonique et de Nanotechnologie, Université de Lille, UMR CNRS 8520, 59650, Villeneuve d’Ascq, France; Institute of Structural Mechanics, Bauhaus-Universität Weimar, Weimar, D-99423, Germany; Department of Geotechnical Engineering, College of Civil Engineering, Tongji University, 200092, Shanghai, China

**Keywords:** 2D materials, hierarchical structure, inverse design, machine learning, metamaterials, phononic crystals

## Abstract

With the growing interest in the field of artificial materials, more advanced and sophisticated functionalities are required from phononic crystals and acoustic metamaterials. This implies a high computational effort and cost, and still the efficiency of the designs may be not sufficient. With the help of third-wave artificial intelligence technologies, the design schemes of these materials are undergoing a new revolution. As an important branch of artificial intelligence, machine learning paves the way to new technological innovations by stimulating the exploration of structural design. Machine learning provides a powerful means of achieving an efficient and accurate design process by exploring nonlinear physical patterns in high-dimensional space, based on data sets of candidate structures. Many advanced machine learning algorithms, such as deep neural networks, unsupervised manifold clustering, reinforcement learning and so forth, have been widely and deeply investigated for structural design. In this review, we summarize the recent works on the combination of phononic metamaterials and machine learning. We provide an overview of machine learning on structural design. Then discuss machine learning driven on-demand design of phononic metamaterials for acoustic and elastic waves functions, topological phases and atomic-scale phonon properties. Finally, we summarize the current state of the art and provide a prospective of the future development directions.

## 1 Introduction

In recent decades, the revolutionary development of functional materials has provided the ability to manipulate photons and phonons [1], [2], [3], [4], [5]. These functional materials are usually composed of artificial periodic or non-periodic arrangements of units and various ingenious structural designs often makes their properties surpass that of natural materials [6], [7], [8], [9], [10], [11], [12]. The in-depth study of (nano)photonic structures, taking advantage of the progress in nanofabrication techniques, has led a series of fascinating applications based on photonic crystals [13], metamaterials/metasurfaces [14], [15], [16], plasmonic nanostructures [17, 18] and so forth. These typical materials have the ability of manipulating the propagation of electromagnetic or light waves for various functionalities. Similar concepts have been further extended to the field of acoustic/elastic waves. The emergence of phononic crystals and acoustic metamaterials have triggered an upsurge in the on-demand design of acoustic/mechanical devices [19], [20], [21], [22], [23], [24], [25], [26], [27], [28], [29] with specific responses.

Forward and inverse design methods are the two main schemes [30], [31], [32] for the realization of these structures. In the forward scheme, the structural responses are directly obtained through theoretical analyses, simulations and possibly experimental approaches [30, 31]. However, to achieve the required objectives or at least approach the expectation, it could be necessary to repeatedly adjust the structural or material parameters and recalculate the response. This trial-and-error process may become very expensive in view of the increase in the design complexity requirements. Therefore, there is an increasing interest in the alternative scheme of inverse design [32] where the appropriate structures are built through the optimization algorithms in the huge parameter space.

The rapid development of artificial intelligence (AI) has made the inverse design ideas a reality. As part of AI, the main optimization algorithms include simulated annealing [33], [34], [35], genetic algorithm [36], particle swarming optimization [37], and topology optimization [38], [39], [40], [41]. These algorithms are quite mature and have been widely reported for achieving on-demand inverse designs. All of them usually rely on the intermediate results obtained by iterative forward design scheme. Due to the limitation of a fully random search, it is still a major challenge to complete the inverse design under multiple constraints. Since the 1980s, machine learning (ML) has gradually become a dominant paradigm in AI community [42, 43]. The basic unit of the artificial neural network, called M-P neuron, was proposed in 1943 [44]. In 1949, Hebb’s rule was put forward, revealing that the basis of neurons learning and memory is the variable connection strength between neurons [45]. In 1958, Rosenblatt proposed the perceptron model which gave for the first time the learning mechanism of neural networks [46]. However, this model does not contain hidden layers and can only deal with linear separable problems. Multilayer neural network (MNN) was widely improved by the proposition of back-propagation algorithm for neural network training in 1980s [47], but they still remained limited by the amount of data and computing power. During the new century, the deep learning technology grew rapidly after 2006 [48, 49], and a series of algorithms were derived from the deepening of the structure of artificial neural network model, also called deep neural networks (DNN). This was supported by the development of big data science and the improvement of computer performance that provided hardware support. Meanwhile, the development of open source and flexible software platforms, such as TensorFlow [50], PyTorch [51], and Keras [52], made it easy for beginners to build generic deep architectures. The third-wave of AI started to bring profound industrial changes in various aspects of modern society, such as computer vision [53], natural language processing [54], speech recognition [55], AlphaGo [56], robotic controls [57], etc. As one of the most important branches of machine learning, deep learning has become the fundamental route in complex hierarchical feature learning.

**Figure 1:**
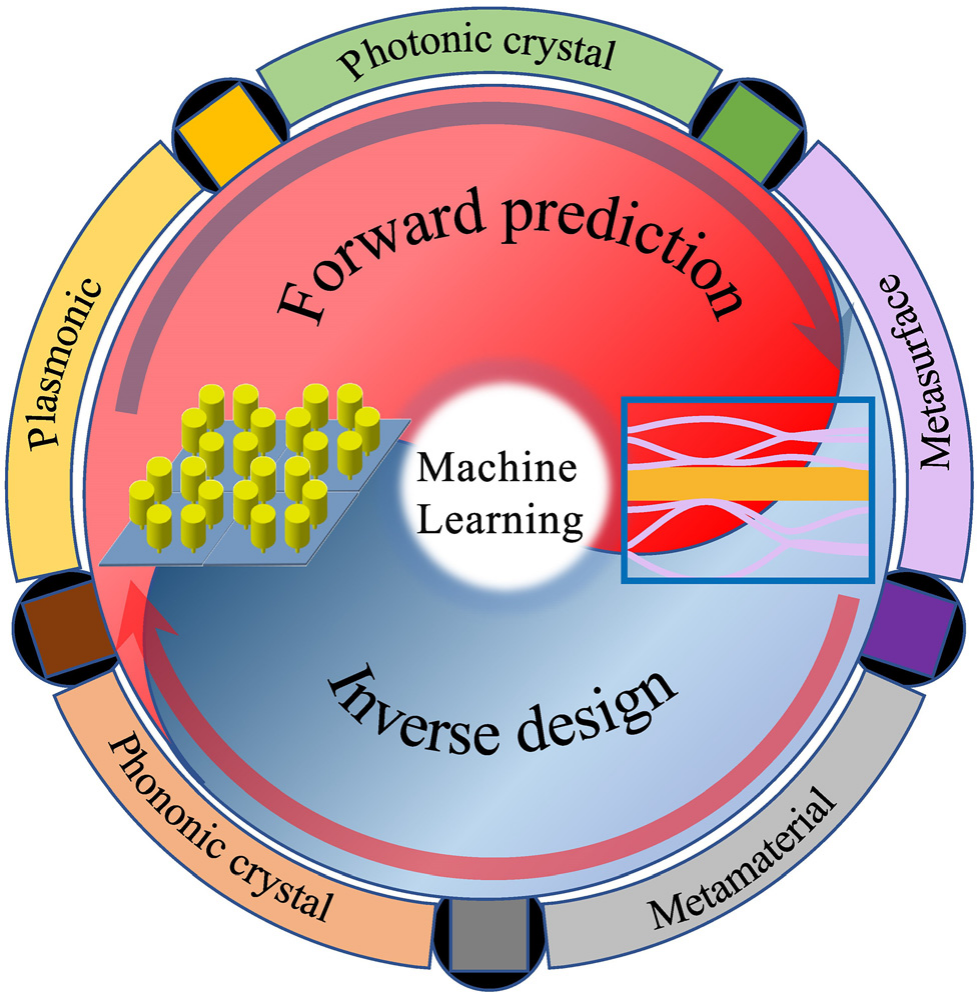
Diagrammatic sketch of machine learning for properties characterization and structural design of artificial materials. The machine learning algorithms are applied to connect the structure (such as phononic crystal, metamaterial, metasurface, photonic crystal and plasmonic) information with the response information (for example, band gap, transmission etc.).

Inspired by the development of AI technology, the intelligent design of materials and prediction of their properties have attracted extensive attention of researchers [58, 59]. The deep integration of deep learning and (nano)photonics has been widely reported in the literature [60], [61], [62], [63], [64], [65], [66]. In this field, DNN can be used to predict the electromagnetic response for a given structure, which is called forward prediction. For example, Peurifoy et al. trained a neural network to approximate light scattering by multilayer nanoparticles [67]. But it is more common to use DNN to inverse design (nano)photonic devices with given electromagnetic responses. However, due to the data inconsistency in the inverse design of photonic devices, that is, one electromagnetic response may correspond to a variety of real structures (one-to-many problem), DNN usually needs to be improved in practical applications. Liu et al. deeply discussed the problem of data inconsistency, and, to overcome it, proposed a tandem architecture neural network combining forward prediction and inverse design [68]. For the tandem architecture neural network, many works have been carried out, such as Objective-Driven all-dielectric metasurface, transmitted metasurface cloak, and so forth [69], [70], [71], [72], [73]. Meanwhile, Malkiel et al. reported a bidirectional DNN to realize both the design and characterization of plasmonic metasurfaces [74]. The bidirectional DNN model was also used by Ma et al. to design and optimize three-dimensional chiral metamaterials that possess strong chiroptical responses with predesignated wavelengths [75]. Excepted the above training modes, some advanced neural networks are also applied in (nano)photonics, such as auto-encoders [76], [77], [78] and generative adversarial networks (GANs) [79, 80]. Different from the direct application of neural network, another application is relying on the training mechanisms of neural network in analogy to physical mechanisms. For example, Lin et al. established an all-optical diffractive DNN architecture [81]. The mature application of ML in the fields of electromagnetic and light waves naturally stimulates researchers’ interest in using ML in acoustic and elastic waves. It should be noted that, from a mathematical point of view, there is no fundamental difference between the two types of waves which are described by similar differential equations. Therefore, many ML algorithms can be successfully extended to the design of phononic crystals and acoustic/elastic metamaterials. As an advanced technology, ML is a powerful tool in the field of artificial material properties characterization and structural design. This is schematically sketched in Figure 1.

In the present review, we discuss the recent advancements in the design of phononic crystals and acoustic/elastic metamaterials based on ML. In Section 2, we introduce the basic principles of the mainstream algorithms for the design of phononic metamaterials. Section 3 focuses on advanced works in the literature to merge ML into acoustic and elastic wave systems. In Section 4, we briefly summarize the ML applied to atomic-scale phononic metamaterials. Finally, we illustrate the prospects and challenges of merging ML and phononic metamaterials in Section 5.

## 2 Principles of ML for phononic metamaterials

ML became an autonomous field of research in the 1980s, and entered an era of maturity and large-scale development in the 1990s and early 21st century. During this period, many algorithms and theories have been developed and applied to a certain extent. Almost at the same time, the concept of phononic crystals was proposed in analogy to photonic crystals and started to grow rapidly [82, 83]. One basic characteristic of phononic crystals is the existence of band gaps caused by Bragg scattering mechanism, which is based on the destructive interference of waves by periodically arranged scatters in space. Different from this mechanism, Liu et al. proposed the local resonant band gap mechanism in 2000, which is a low-frequency hybrid band gap formed from the avoided crossing of two bands with the same symmetry [84]. The mechanism of local resonant band gap led to the development of acoustic metamaterials. Although the development timelines of ML and phononic metamaterials almost overlap, the merger of these two areas occurred with a certain time lag. In this section, we introduce the mainstream ML algorithms that have been applied to the field of phononic metamaterials.

### 2.1 Supervised learning

**Figure 2:**
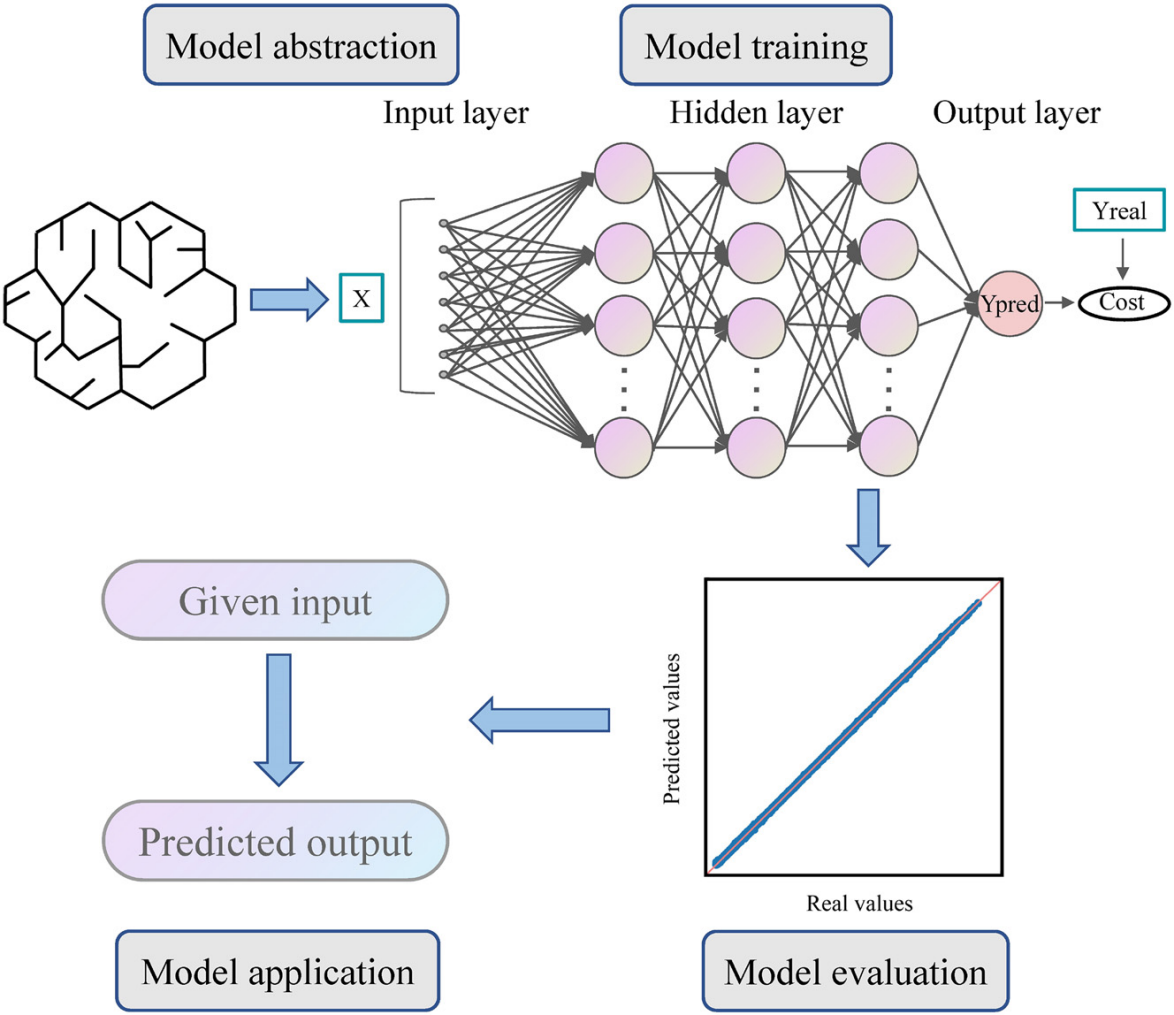
Working principle of artificial neural networks. The neural network model is inspired by the nervous system of the brain and consists of an input layer, a hidden layer and an output layer. The model is trained and evaluated with the previously obtained dataset. Once the model is trained and evaluated, it can be employed to properties characterization and structural design of artificial material.

According to the learning mode, most ML algorithms can be classified as supervised learning, unsupervised learning and reinforcement learning [85]. The most common form is the former one presented in this section [48]. For the artificial neural network [86] in Figure 2, the model is based on the simulation of brain mechanisms and is the product of connectionism in AI [87]. In this model, many artificial neurons map the input layer data to the output layer by layer connection; there are connections between any two neurons in adjacent layers, so the process is also called fully connected neural network. The training samples of supervised learning are composed of eigenvectors (*x*) and labels (*y*). Supervised learning can be generally divided into two types of problems: classification and regression. The mechanism is to approximate an unknown function *f* with neural networks *y* = *f* (*x*; *θ*), where *θ* represents the connection weight and biases between layers, which needs to be determined through continuous training.

In 1986, Rumelhart et al. [47] proposed the famous back-propagation algorithm, which solved the problem of hard training for MNNs, and began to be widely used for practical problems. During training, a cost function is defined to quantify the discrepancy between the network output and the desired output. The goal of training is to minimize the cost function by using the gradient descent method, and update all weights and biases layer by layer. The learning progress of the model is controlled by some hyperparameters. For example, the learning rate is the update step of the weights in each iteration of the algorithm. The traditional gradient descent algorithm is very sensitive to the learning rate, and the problem is more prominent in high-dimensional space and MNN. Specifically, if the learning rate is too low, the search speed is slow, and if it is too high, it may skip the extreme point. Meanwhile, the algorithm can easily get stuck at the saddle point in the iterative process due to the fixed learning rate. Therefore, some adaptive optimization algorithms, such as AdGrad, RMSProp and Adam, have been developed to overcome this problem. In these optimizers, the learning rate is no longer a fixed value, but is automatically adjusted as the iteration progresses according to some specific rules.

After training, the unseen test samples in the training are usually used for model evaluation. Finally, the well-trained model can be applied for predictive modeling. From the mathematical point of view, neural network is a composite function, and its nonlinear information processing ability is provided by the nonlinear activation function of neurons. The universal approximation theorem shows that if the activation function is properly selected and the number of neurons is sufficient, the neural network with one hidden layer can be used to approximate any continuous mapping function from input vector to output vector [88]. The ability of neural network to capture the nonlinear characteristics of high-dimensional space makes it powerful to capture the complex nonlinear relationship of structures–properties (or properties–structures).

In addition to fully connected neural network, convolutional neural network (CNN) [89] is also widely used in artificial material design. The hidden layers of CNN include convolution layers, pooling layers, full connected layers and normalization layers. Convolution operation is performed on the input data to extract features, and then the feature selection and information filtering are carried out through the pooling layer. After repeating extraction and selection filtering, the output is obtained through the full connected layer or global average pooling layer. CNN possesses advantages in capturing image information, which can efficiently map the field images information to the corresponding structures, or the 2D structure images to the responses. As the internal connections of CNN are much fewer than those of the standard model with the same depth, it can not only reduce the difficulty of training, but also ensure that more complex mappings can be realized.

### 2.2 Unsupervised learning

The unsupervised learning analyzes the unlabeled samples and finds the structure or distribution law of the sample set. Clustering and data dimensionality reduction are typical representative methods. The goal of clustering is to directly divide the sample set into multiple classes without training process. Clustering is essentially a set partition problem. Because there is no manually defined category standard, the key issue of clustering is how to define different classes. Therefore, many clustering algorithms have been derived from different class definitions, such as those based on centroid, probability distribution and density, etc. High dimensional data can be mapped to low dimensional space through linear or nonlinear dimensionality reduction techniques such as principal component analysis (PCA) and manifold learning, which can be easier for analysis and clustering.

**Figure 3:**
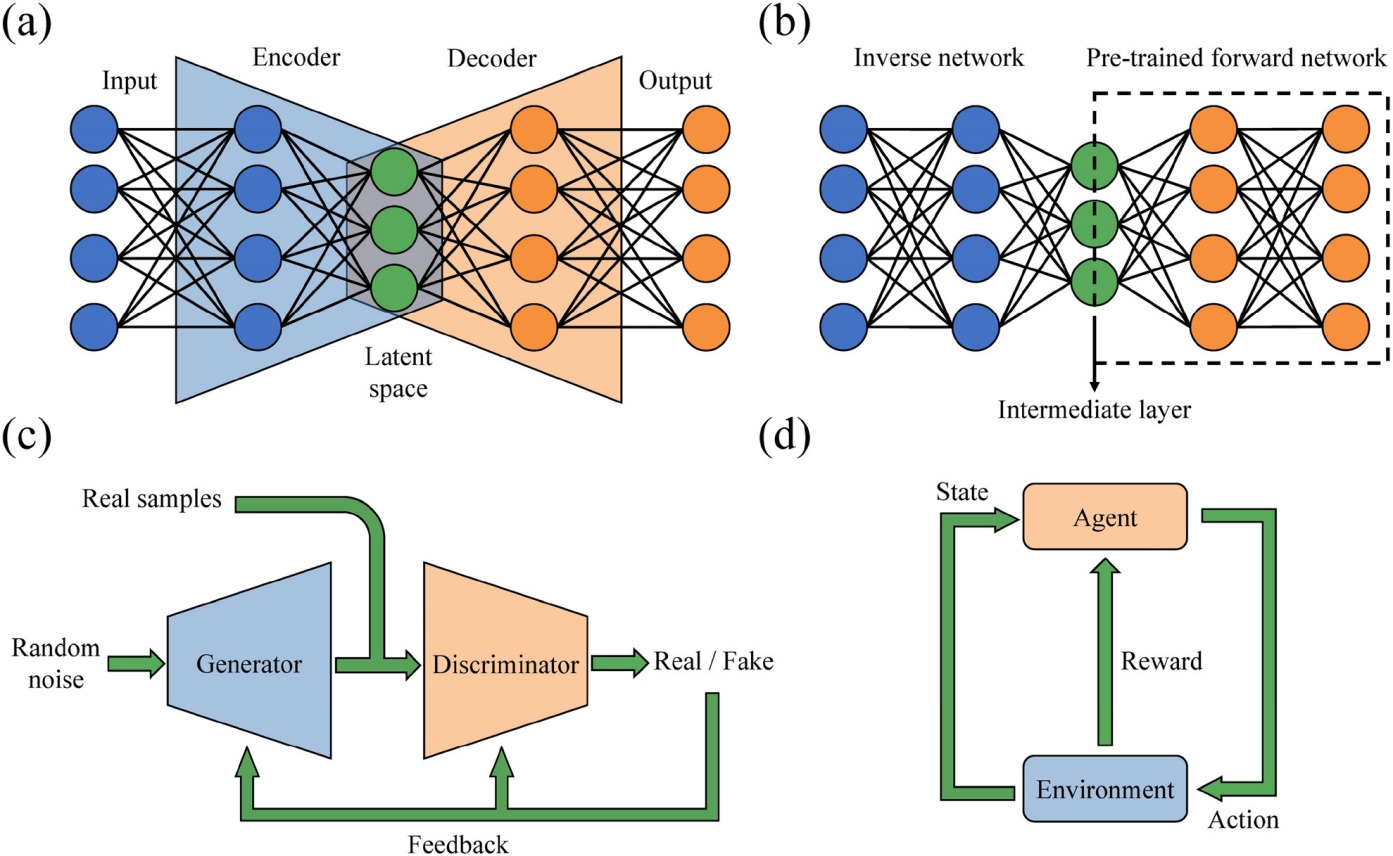
Schematic diagrams of unsupervised learning and reinforcement learning. (a) AE. The encoder maps the high-dimensional data to the low-dimensional latent space, and the decoder can reconstruct the high-dimensional data according to the features. (b) TNNs. An inverse design network connected to a forward modeling network. The forward modeling network is trained in advance, then train the whole network under the condition of freezing the weights in the forward modeling network. The design results are output from the intermediate layer. (c) Generative adversarial networks. The generator and discriminator compete with each other, so that the generator has the ability to output close to the real samples. (d) Reinforcement learning. The agents execute appropriate actions in the environment to obtain the maximum cumulative reward.

Auto-encoders (AE) is a special DNN structure, which can extract features and reduce data dimension with the help of the powerful nonlinear processing ability of neural network. The first part of AE is the encoder, which is used to extract features from the original input data, and the second part is the decoder, which reconstructs the original data according to the extracted features, as shown in Figure 3(a). The input data successively passes through the encoder and decoder to obtain the output vector, and then calculate the reconstruction error from the comparison of the output vector and the original input vector. The reconstruction error is defined as the cost function, and employ back propagation algorithm to minimize it. Tracing to the source, the AE uses the original input data as labels instead of the sample labels in the dataset, so it belongs to unsupervised learning. The latent space vector contains the featured information of the reduced dimension of the input data, which can be used for further visualization or analysis.

The tandem neural network (TNN), which is widely used in the design of artificial materials, is developed based on the idea of AE. TNN is composed of an inverse network connected to a pre-trained forward network, as shown in Figure 3(b). The forward network which is trained in advance takes the design parameters as the input and the responses as the output. Therefore, the training for forward network is under supervised conditions, and because one structure corresponds to only one response (one-to-one problem), the training process is easily converged. In the training of the whole network, the weight and bias of the forward network are fixed, and the weight and bias of the inverse network are adjusted to reduce the cost. The cost function consists of the discrepancy between the predicted responses and the target responses. When using TNN for inverse design, the desired responses are taken as input, and the corresponding design structures obtained from the output of the intermediate layer. In the training process of the whole network, the input responses are also used as labels, so this step can be regarded as unsupervised training. Compared with AE, the training process of TNN is more cumbersome, but the model can directly connect the responses and structures instead of extracting features. More importantly, it overcomes the one-to-many problem in inverse design, as discussed in Section 1. To process image data, the fully connected network in AE or TNN can be replaced by CNN, which is only a computational replacement without changing the basic principle.

GAN [90] is another promising algorithm for unsupervised learning in material design. GAN consists of a generator and a discriminator, as shown in Figure 3(c). The generator takes the random noise as input, which is usually realized by MNN, and provides generated samples at the output. These generated samples will be fed into the discriminator together with the real samples. The discriminator is a binary classifier and judges whether these samples are real or generated samples, and feeds back the judgment results to guide the model update. Therefore, the task of the generator is to learn the probability distribution of the real samples and make the generated samples as similar as possible to the real samples to deceive the discriminator. While the task of the discriminator is to distinguish real and fake samples as accurately as possible. During training, the two models are optimized alternately and compete continuously until the classification accuracy of the discriminator is 0.5, which indicates that the discriminator cannot distinguish whether the samples are real or fake, and the system achieves balance. For application, the generator in the well-trained GAN can be taken out separately to generate samples. However, this conventional GAN model can only generate samples similar to the real samples according to the distribution, and cannot generate the corresponding samples according to the expectation. Therefore, to be applied to the inverse design of materials with desired response, a variant of GAN model is usually used, which is called condition generative adversarial networks (CGAN) [91, 92]. In addition to inputting random noise into the generator, CGAN can also attach the characteristics of samples as conditions. Thus, the CGAN generates the desired generated samples more accurately. In this model, we can combine responses and random noise as input. Meanwhile, the real samples should include the structures and corresponding responses. The training process is consistent with the conventional GAN model. When using the generator, we can generate the corresponding structures by inputting the desired responses and random noise, which is an inverse design process. It should be noted that, due to the use of both structure and response in CGAN training samples, there is a fuzzy classification of whether CGAN belongs to supervised learning or unsupervised learning. But it is undeniable that CGAN is an elegant scheme of material inverse design. Furthermore, the model is not limited to fully connected networks, but can also use CNN and even other ML models, as long as it conforms to the framework of GAN [93, 94].

### 2.3 Reinforcement learning

Reinforcement learning [95] is a kind of ML algorithm based on environment interaction inspired by behaviorism psychology. Different from supervised and unsupervised learning, reinforcement learning does not need to prepare training samples in advance. Its goal is to learn how agent executes actions in the environment to obtain the maximum cumulative reward. The agent selects and executes an action in the current state, and then enters to the next state, and the environment will feed back a reward for the current action, as shown in Figure 3(d). The system updates and stores the value function of the current state according to the reward, to force the agent to execute correct action in the same state in the future. SARSA [96] and *Q*-learning [97] are typical representative algorithms. Their state value function is called *Q* function, and stored in *Q* table, which is a two-dimensional table. The difference between the two algorithms is the way they update the *Q* table, namely the former updates the *Q* value corresponding to the next action, which is called on-policy learning, while the latter only updates it by choosing the current action, which is called off-policy learning.

When reinforcement learning is used in material inverse design, it only needs to clearly assign what the agent and environment are. One reasonable case is to extract the optimization objective from the response and define it as the environment, while the structural or material parameters are defined as the agent. The parameters execute actions to increase or decrease themselves to achieve the optimization goal by changing the response. In this way, the trained model can determine the structures corresponding to the optimization goal. In the practical applications of designing complex structures, we may encounter the problem that the number of state value functions is too large to be stored in arrays. This problem can be solved by deep reinforcement learning, which is an algorithm combining DNN and reinforcement learning. For instance, employ the neural network to approximate the state value function, with the states as the input and the output are the function values of various actions, this algorithm is called Deep Q networks (DQN) [98]. DQN greatly promotes the storage capacity of the system and is an effective means to enforce the on-demand design of more complex structures.

When selecting the above algorithms, we usually pay great attention to the two factors of computational accuracy and computational cost. Both supervised learning and unsupervised learning belong to data-driven methods, and their computational accuracy largely depends on the size and distribution of data. Meanwhile, the reasonable selection of hyperparameters will also have a certain impact. Therefore, when aiming at a specific problem, it is necessary to obtain the dataset according to the complexity of the problem, and check the distribution of the data before it is used for model training. In the training process, the cross-validation method is usually used to select the optimal hyperparameters. As for the calculation cost, it depends on the amount of data, the structural complexity of neural network model, the selection of activation function and so on. Reinforcement learning does not need data training, but gradually approaches the preset goal through iteration. The accuracy of its calculation also depends on the selection of hyperparameters to a certain extent. The key to improve the accuracy is to avoid falling into local optimum through appropriate exploration step length selection. The computational cost usually depends on the degree of discrepancy between the initial state and the target state.

## 3 Design of phononic metamaterials enabled by ML

The solid foundation achieved in the flexible design and application of MLs in (nano)photonics provides a valuable framework for developing the understanding of MLs in acoustics and mechanics. Applying ML algorithms to the property’s characterization and inverse design of phononic metamaterials is an efficient means to precisely control acoustic and elastic waves toward the achievement of singular properties. We have introduced the basic principles of ML in the design of phononic metamaterials in the previous section. Here, we focus on the recent advances of the specific applications of these algorithms.

### 3.1 ML for acoustic metamaterials

**Figure 4:**
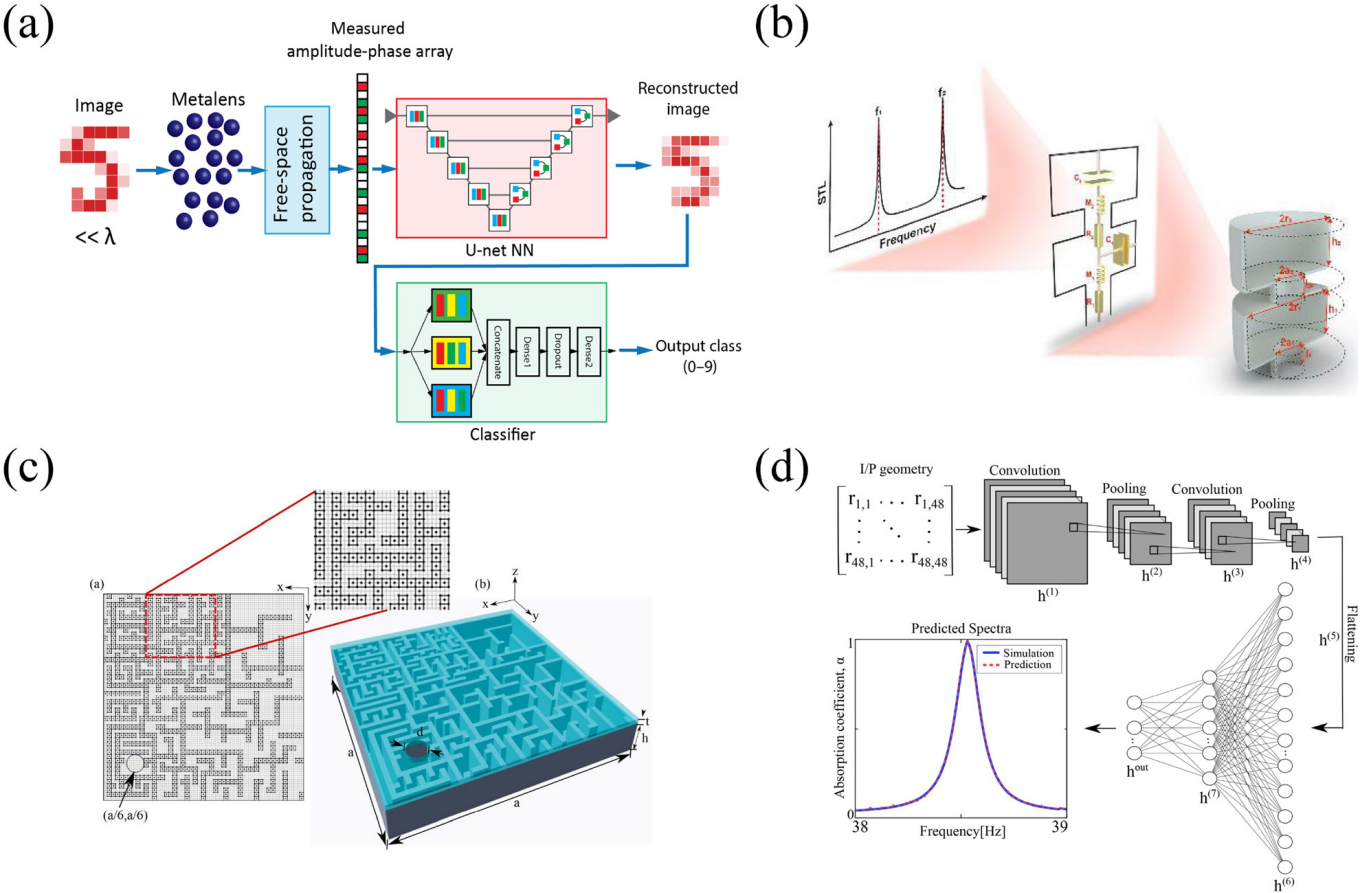
Properties characterization and inverse design of acoustic structure based on deep learning. (a) Breaking the diffraction limit and realizing far-field subwavelength acoustic imaging [99]. (b) Inverse design of multi-order Helmholtz resonators using DNN and LPT [100]. (c) The metasurface absorber and matrix coding [101]. (d) Predicting absorption spectra according to coding matrix by CNN [101].

It is well known that the imaging enables us to observe and recognize objects by analyzing light and sound waves transmitted or radiated by them. But the existence of diffraction limit makes it difficult to restore all details of the image in the far-field. This is because the evanescent waves scattered from the subwavelength regions of the object cannot propagate to the far-field, resulting in a loss of information. More recently the progress in metalens structures has greatly promoted the development of super-resolution imaging technology. However, the absorption loss of metalens is often large, which greatly limits their application. Orazbayev et al. combined deep learning with metalens technology to efficiently realize far-field subwavelength acoustic imaging [99]. More importantly, the absorption loss greatly improves the imaging effect in this method rather than inhibiting it. The scheme of subwavelength far-field acoustic imaging based on deep learning is shown in Figure 4(a). A metalens composed of a cluster of randomly placed subwavelength Helmholtz resonators is inserted in the near field of subwavelength acoustic source to couple the evanescent field components and reradiate the waves into the far-field patterns. The amplitude and phase of the far-field acoustic waves are measured by a microphone array. Then a “U-net-type” convolutional neural network (UCNN) is trained to connect the far-field amplitude and phase patterns with the subwavelength images. Tracing to the source, the proposed UCNN model is an AE with convolutional layers, which realizes the function of reconstructing images from far-field acoustic information. The reconstructed images are classified using a standard CNN to identify the numbers in the images. Because the resonance effect of the metalens near a given frequency enhances with the increase of loss, the effective mode density will increase, which can increase the efficiency of the neural network to extract high-resolution imaging information from the resonance mode.

The algorithm of the above framework reconstructs the structure from the amplitude and phase information of the radiated acoustic waves. To be usable in a variety of cases, it is necessary to design the structure from the complex response spectra. In what follows, we introduce a case of designing structures according to the sound transmission loss spectra (STL). A Helmholtz resonator is an easy-to-design acoustic structure with the functionality of manipulating low-frequency acoustic waves. Multi-order Helmholtz resonators can provide multi-order resonances and realize richer airborne applications. In practical applications, it is a great challenge to inverse design the structures according to the desired STL. The study of Sun et al. shows that this difficulty comes from the process of inferring equivalent electrical parameters (EEPs) from the desired STL spectrum which requires solving a six-degree equation for the resonant frequencies [100]. Indeed, the knowledge of EEPs allows easily accessing the geometrical parameters (GPs) by using lumped-parameter techniques (LPT). To solve this issue, they trained the DNN to realize the mapping between STL and EEPs, to avoid solving higher-order equations. It is worth noting that the forward process from GPs to EEPs and STL is easy to implement, so the dataset can be obtained by analysis. In general, this ingenious inverse design process is divided into two steps (see Figure 4(b)): (1) obtain the EEPs by DNN with the desired STL spectrum. (2) Obtain the GPs by LPT with EEPs.

Different from inverse design, forward properties prediction can usually be realized by analytical or numerical methods, the latter being often necessary for some complex structures. In this case, the role of ML is not to solve the impossible or difficult tasks, but to surpass the efficiency of numerical methods by reducing the time-consuming calculations. Donda et al. applied CNN to accurately predict the absorption spectrum of a given metasurface [101]. In this work, the metasurface consists of randomly curled patterns inside the cavity, as shown in Figure 4(c). The total lattice is encoded into a matrix where 0 and 1 represent air and polylactic acid (PLA) respectively. To obtain dataset for neural networks training, they calculated the absorption spectra of some randomly generated coding modes by finite element method (FEM). Under the training and testing of these small amounts of data, CNN can accurately and generically predict the absorption response of all coding modes (see Figure 4(d)). Their work shows that this absorption spectrum prediction scheme is more than four orders of magnitude faster than conventional FEM, saving considerable time and computational cost.

**Figure 5:**
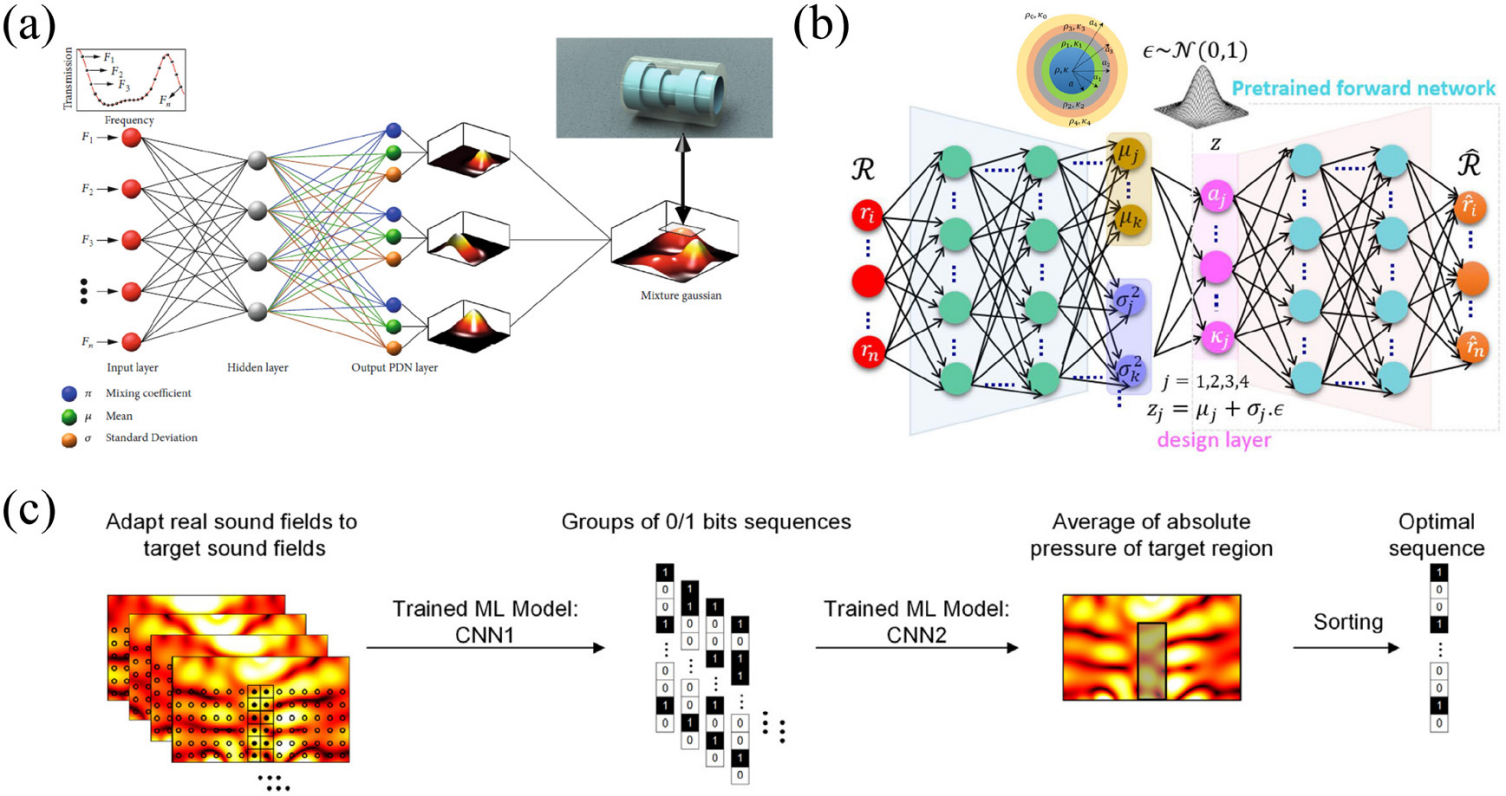
Probabilistic machine learning inverse design scheme. (a) Fuzzy design for acoustic structures combining deep learning and Gaussian mixture sampling [102]. (b) Design of core–shell acoustic cloak based on probabilistic TNN architecture [103]. (c) Machine learning for controlling regional sound field [104].

Due to the one-to-many problem between response and structure, the conventional neural network usually cannot design the structure correctly. A very intuitive way is to introduce a probabilistic approach to select the most appropriate structure among the candidates. One of the representative work is done by Luo et al. [102], who proposed a hybrid architecture combining deep learning with mixture Gaussian sampling, as shown in Figure 5(a). The front end of the model is a DNN that maps the target transmission spectrum to individual Gaussian distribution parameters. The Gaussian distribution parameters include mixing coefficient (*π*), mean (*μ*), and standard deviation (*σ*), which are combined with input *x* and target label *y* to form a special loss function for neural network training. The rear end receives parameters output by neural network and linearly overlays all the individual Gaussian distributions to form a mixture of Gaussian distributions. The local maxima of the mixture Gaussian distribution correspond to suitable candidate structures. Experiments show that the transmission spectra of these candidate structures are basically consistent with the target. This method starts from a fuzzy design idea, which can provide several suitable candidates close to the target, rather than a single solution.

Another example of probabilistic deep learning model is reported by Ahmed et al. [103], their design goal being a cloak with a multilayered core–shell configuration. In their probabilistic model based on TNN model, the front end transforms the input spectral response into a mean vector and a variance vector in order to approximate the standard Gaussian distribution of the latent variables corresponding to the design space instead of mapping directly to design space, while the rear end is still a forward pre-training network (see Figure 5(b)). The design parameters of the structure are obtained by sampling from the Gaussian distribution. The probabilistic model has good tolerance for design faults and enhances the generalization ability.

The two cases above provide a variety of options for structure selection, which is in line with reality, but often select the structure with the highest degree of matching with the desired response, and the other candidates are rejected. Achieving local enhancement or attenuation through sound field regulation is one of the important topics in acoustics research. The degree of enhancement or attenuation should generally be determined on the basis of actual engineering or environmental needs, which allows all candidates designed by ML to have the actual working conditions. Zhao et al. proposed an ML optimization method to design the phase gradient of the acoustic metasurface for control of the local sound field [104]. The phase gradient of metasurface is digitized into 0/1 bit sequence, and the sound field is calculated by FEM to generate dataset. CNN1 is trained to realize the mapping between the absolute sound field and the metasurface sequence, while CNN2 is trained to realize the mapping between the metasurface sequence and the average sound field. A group of adaptive sound fields are input into CNN1 to obtain a series of metasurface sequences, and then the average value of sound pressure field in the target region can be obtained through CNN2 (Figure 5(c)). By sorting the average values, the best sequence to realize the desired sound field intensification or weakening can be found.

**Figure 6:**
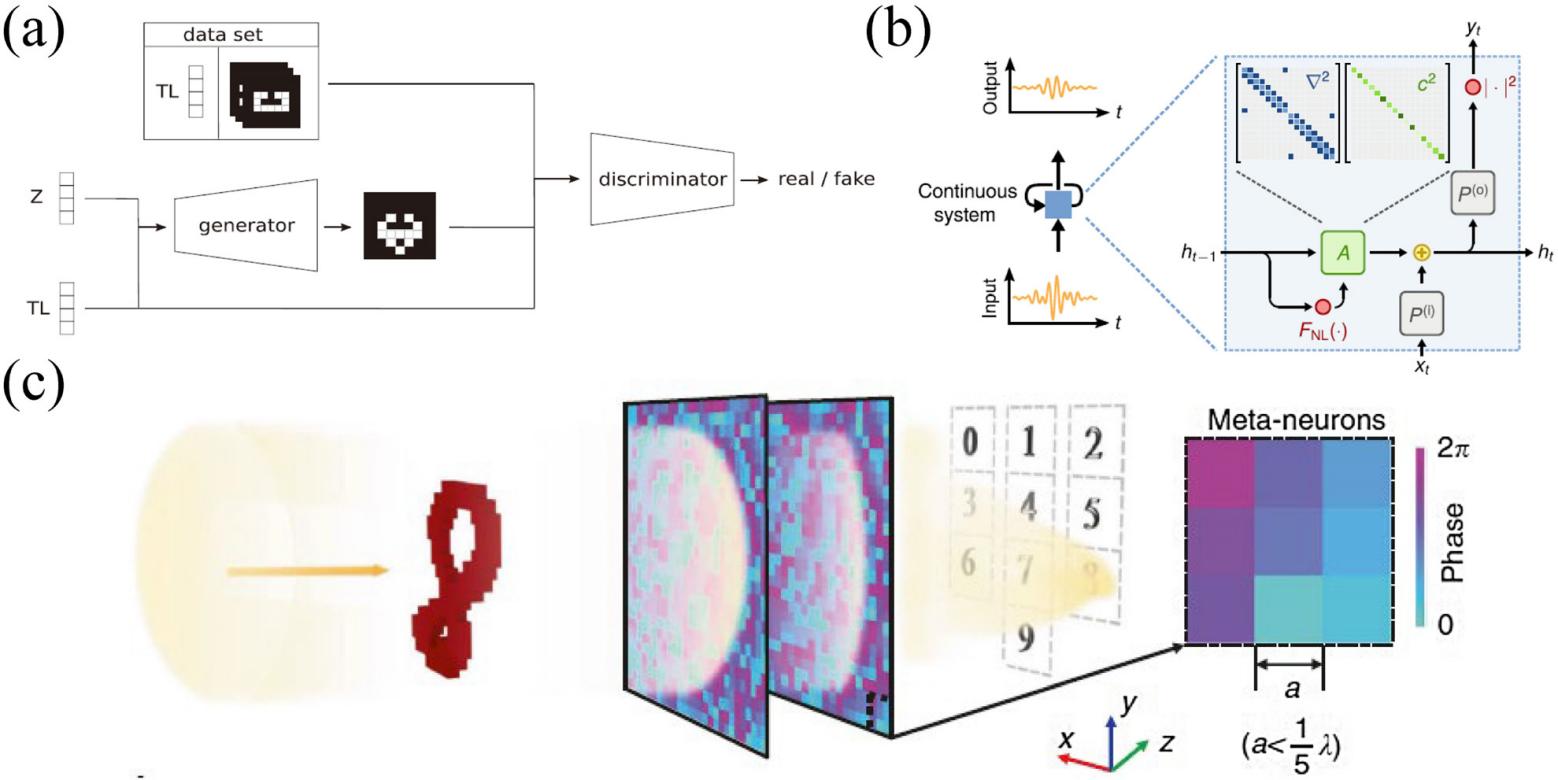
Advanced generative models frameworks in acoustic metamaterials and simulation of wave physical mechanism with neural network model. (a) Designing acoustic metamaterials based on generative adversarial networks [105]. (b) The analogy of acoustic wave physics system and RNN [106]. (c) The analogy of acoustic meta-neural-networks and CNN [107].

For the inverse design of structures with complex desired responses, the idea of deep generative model has been proposed which is completely different from all of the previous frameworks. Some applications have been reported in the field of acoustics. For example, Gurbuz et al. developed a method to design acoustic metamaterials based on CGAN [105], in the frame shown in Figure 6(a). They used a random algorithm to generate binary images of the unit cells, which is composed of fluid elements and solid elements, and obtain the transmission loss spectra through FEM. The generator generates images based on random noise and transmission loss spectrum. The generated images and the real images are mixed together and input into the discriminator for judgment. Complete training is achieved through competition between generator and discriminator. This work successfully used CGAN to find the underlying relation between transmission loss and cell geometries, and achieved the inverse design of the structural unit cells for the desired sound insulation purpose. It deepens the understanding of the application of generative model in the field of acoustics, and is of some pioneering significance.

The works we introduced above are the direct application of ML algorithms. As discussed in the introduction, another application of ML in the field of artificial materials is training mechanism simulation and extension of neural networks, which is an indirect application. More specifically, by constructing real physical structural units and imitating the training method of neural networks, one can train real units to realize waves control in reality. The indirect application is based on the fact that wave propagation in phononic metamaterials is similar to data propagation in neural networks, which gives them similar mathematical expressions. Hughes et al. confirmed that the dynamics equation of wave physics is similar to the computation in recurrent neural network (RNN) after certain mathematical processing [106]. This means that standard training techniques of neural networks can be used to train wave systems to learn complex features in temporal data, the physical analog model being shown in Figure 6(b). To prove the equivalence, they discretized the second-order partial differential governing equation of scalar wave field in time, and obtained expressions similar to the data propagation in RNN. As a demonstration, they showed that the inverse designed inhomogeneous medium can classify the original audio signals with vowels, achieving performance comparable to the standard digital implementation of RNN. Another novel mechanism simulation is reported by Weng et al. [107]; they first proposed and verified the acoustic meta-neural-network through the theoretical derivation and experiments. The units of this neural network are constructed of metamaterials, called meta-neurons. Their theoretical derivation shows that the relationship of scattering wave propagation between layers of meta-neural-network is similar to that of conventional neural networks. Therefore, the training of meta-neural-network can be realized by iteratively adjusting the whole phase profile of meta-neurons in each layer. Furthermore, they experimentally verified the application of meta-neural-network in handwritten digit recognition and the recognition of orbital angular momentum beams. Due to the subwavelength characteristics of metamaterials, meta-neural-networks can be miniaturized and compact in design, and thus having certain advantages in acoustic applications. The conceptual connection between physics and ML will pave the way for a new simulation hardware platform.

### 3.2 ML for elastic metamaterials

The works reviewed above represent the fusion of ML in the field of acoustic metamaterials. In this section, we review some recent attempts to make a fusion of ML in the field of elastic metamaterials. Dispersion relations which result from the periodicity of the structure are the main theoretical basis for the study of phononic crystals. Finol et al. proposed using neural network to approximate the input and output of eigenequations for simulating the nonlinear relationship [108]. They studied the eigenequations of 1D and 2D phononic crystals, and used CNN to map the mechanism between material property parameters and eigenvalues. Their work shows that the nonlinear modeling ability of neural network can be used to solve mechanical eigenvalue problems. Inspired by this, Liu et al. employed neural network to predict the dispersion relationship of 1D layered phononic crystals [109]. Based on the above basis, they further proposed to use supervised neural network (S-NN) and unsupervised neural network (U-NN) to inverse design this structure [110]. Actually, the U-NN is TNN, which is discussed in Section 2 and is more accurate than the conventional S-NN in the case of multi parameters inverse design.

**Figure 7:**
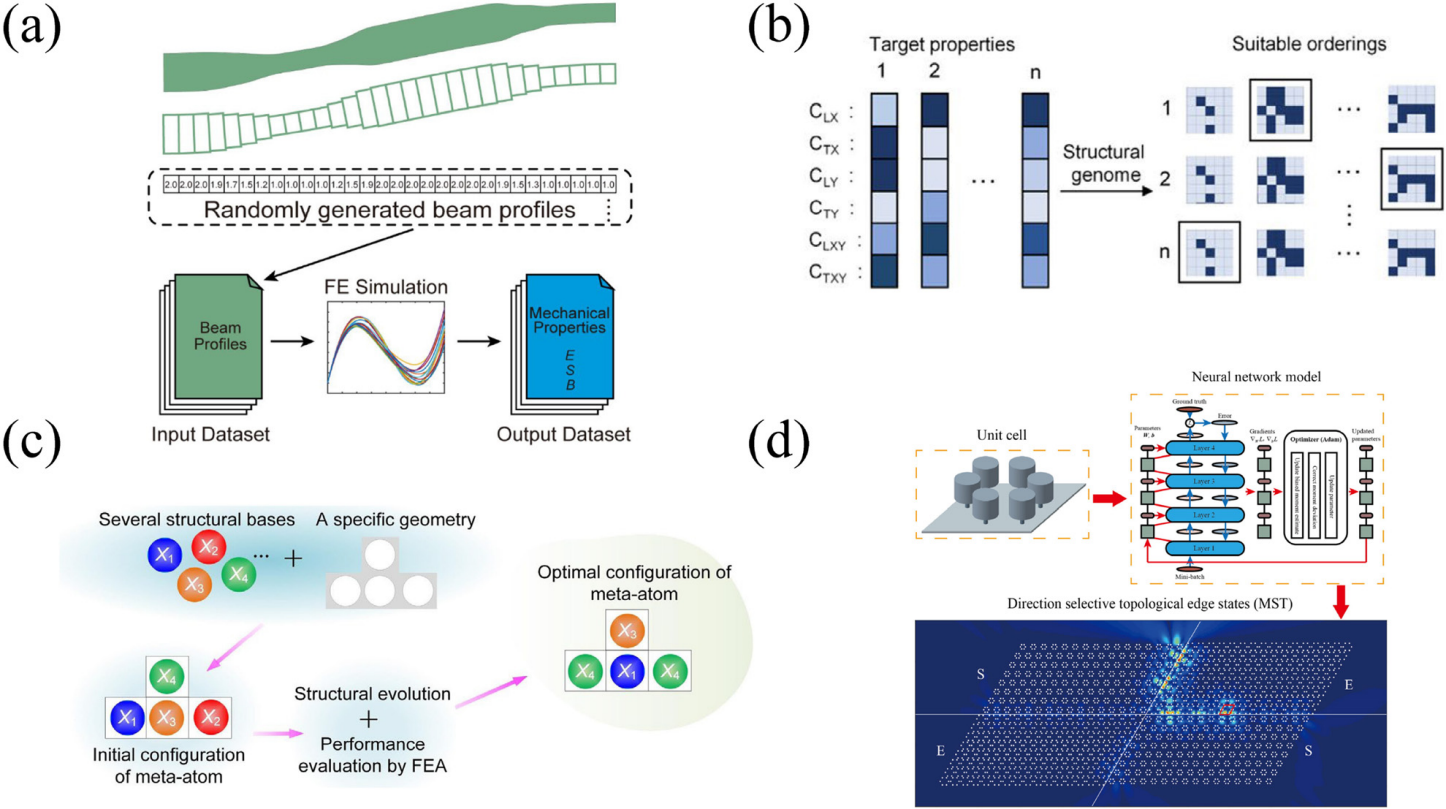
Applying neural networks for elastic metamaterials design. (a) Design of curved beams with variable thickness [111]. (b) Constructing structural genome of digital coding metamaterials by neural network to realize rapid structural screening for specific wave velocity properties [112]. (c) Optimizing configuration of meta-atom to obtain modular metamaterials with specific properties [113]. (d) Design of phononic thin plate with specific bandgap width to realize highly robust elastic wave transmission [120].

The mechanical beam is another 1D system, which is a basic structural platform of mechanics and has rich practical application scenarios. Compared with traditional straight beams, curved beams with variable thickness have richer characteristics, such as the bistability, but the design process is more difficult. Liu et al. demonstrated a new method to realize the design and optimization of variable thickness curved beams via ML [111]. The mechanical properties of curved beams such as stiffness (E), forward (S) and backward (B) snapping force can be adjusted by changing the thickness distribution of the beam. They randomly generated the thickness distribution of the curved beam and obtained the corresponding mechanical properties through FEM, Figure 7(a). Through the above steps, they generate a dataset for DNN training to capture the complex nonlinear relationship between the variable thickness curved beam and its mechanical properties. According to the requirements of different mechanical properties, an optimization step is implemented to obtain a high-property curved beam. Their work applied ML algorithms to efficiently and accurately design curved beams, which shows the suitability of ML for mechanical metamaterial structure design.

Digitally coded metamaterials achieve different properties by changing the coding order of representative volume element (RVE) rather than the geometric structure. Generally, the RVE has two different materials, represented by 0/1 bits, which introduces a new degree of freedom and contains a large number of possible combinations. Because the encoding order and the response cannot be linked by an analytical expression, and the numerical method is quite time-consuming, it is a challenge to determine the coding order according to the required properties. A natural idea of building a huge digital structured genome with all possible sequences has been proposed by Zhang et al. [112]. Their digital materials have a total of 2^25^ kinds of 5 × 5 layout composed of 0/1 bits. The dispersion curves of specific structures are obtained by FEM, from which the wave velocity properties could be extracted. They used FEM to produce a certain number of samples as the training set, and trained the CNN to find the mechanism between the digital material structures and the wave velocity properties. The well-trained neural network model can replace FEM, take all possible structures as inputs in turn, and efficiently find the corresponding wave properties. In this way, a huge genome containing 2^25^ samples can be established. The digital structural genome provides an efficient way to find digital materials in genome with the most suitable orderings based on target properties, see Figure 7(b).

Similar to digital coded metamaterials, modular metamaterials also realize the properties of the overall structure through different combinations of internal units. Wu et al. proposed a design scheme for modular metamaterials based on ML [113]. In this scheme (see Figure 7(c)), some basic components are selected from the existing studies to construct an initial configuration of meta-atoms. Then, the structure evolution is realized by genetic algorithm (GA) or neural network until the meta-atoms conforming to the specific properties are found. For the GA, a few iterations are often sufficient to find the best configuration. Compared with traversing all possible configurations, the computational cost of FEM is significantly reduced. For the neural network, the dataset for training is obtained by FEM according to a certain number of configurations. With a well-trained neural network, the best configuration that meets the specific properties can also be found among all configurations. The authors provide four cases to illustrate the implementation of the scheme. The results show that the scheme can make full use of the existing knowledge of metamaterial design and realize the modular metamaterial design with specific target properties with the help of ML.

Different from the mechanical properties realized in the above work, the development of non-Hermitian acoustics and topological acoustics has greatly enriched the physical properties of phononic metamaterials [114], [115], [116], [117], [118], [119]. In particular, the existence of topological edge states at the interface of two topologically different phases is demonstrated such states show high robustness against disorder and imperfections in the structure. However, the search of mechanical wave state at a specific frequency and bandwidth depends on the accurate inverse design of the structure. Some of the authors employed ML algorithm to inverse design topological metaplates with resonators and realized strongly robust topological edge states [120]; the design process is illustrated in Figure 7(d). In our design, the dataset is obtained by plane wave expansion (PWE) method, and the trained neural network can predict two different geometric structures for the same bandgap width, which are shrunk type and expansion type. The edge state can be obtained in this common bandgap frequency range. For the interface formed by two structures, we confirm that the edge state localization of the wide common bandgap structure is higher than the narrow common bandgap. Moreover, the spin excited edge state shows directional propagation and is able to turn around sharp corner without back scattering effect. Our work shows that the structure design based on ML allows the realization of advanced properties such as topological states.

**Figure 8:**
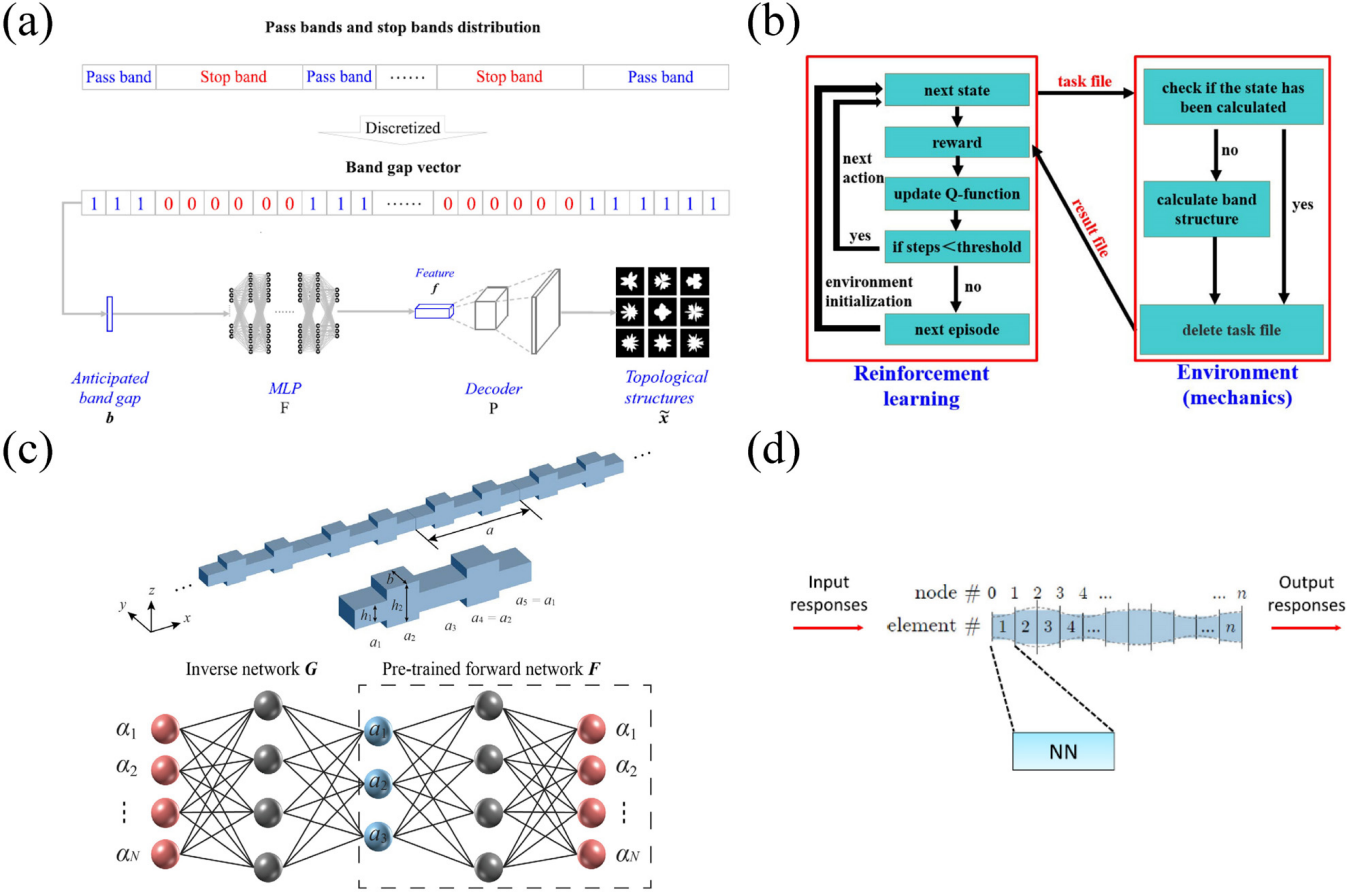
Elastic metamaterials design enabled by advanced neural network model and reinforcement learning. (a) Design of phononic crystals by AE and MLP. The MLP link the band information and geometric characteristics of structures, while the decoder is employed to reconstruct the structures [122]. (b) Design of layered phononic crystals with specific band structures by reinforcement learning [123]. (c) Design of phononic beams with specific bandgap width and topological properties by reinforcement learning and TNNs [124]. (d) Simulation of the structural units using neural networks [125].

Some recent works have also shown the ability of AE in mechanical structure design. For example, Hanakata et al. [121] demonstrated the ability of AE to forward and inverse design graphene structure. A more typical work is reported by Li et al. [122], where AE is employed to design phononic crystals with anticipated bandgaps, as shown in Figure 8(a). The RVE of 2D phononic crystals studied in this work are composed of irregular scattering inclusions in the matrix. The shape of the scattering inclusions can be randomly generated by the analytical function. They first trained an AE based on CNN with a large amount of data to extract the features of the topological structures. Then, they used FEM to calculate the dispersion relations corresponding to the topological structures. On the premise of discretizing the dispersion relations, a multilayer perceptron (MLP) is trained to build the mapping from the bandgap distribution to the topological features (the label obtained from the output of the encoder). Therefore, after training the AE and MLP, the design process can be described such as to obtain the feature by MLP according to the anticipated bandgap, and then restore its topological structure through the decoder. This design scheme is very ingenious, where the encoder is replaced by MLP, while the decoder is retained to complete the feature reconstruction from bandgap information to structure.

Further, this team creatively proposed the application of reinforcement learning in the design of 1D layered phononic crystals [123]. They considered the two thickness parameters of the layered phononic crystals as an agent. The agent gradually explores the influence of increasing or decreasing structural parameters on the band structures during training, and updates experience through environmental feedback to force it to make correct action choices in subsequent training; the process is illustrated in Figure 8(b). By applying *Q*-learning (one of the reinforcement learning algorithms), they realized the design of phononic crystals with the goal of maximizing the bandgap width and customizing the bandgap range. This work demonstrates the powerful ability of reinforcement learning in target mechanical design.

Inspired by this work, our team recently proposed using reinforcement learning to design phononic beams with periodic variable cross-section [124]. We derived the dispersion relation by transfer matrix method (TMM), so that we can analytically obtain the bandgap width. We employ this process as the environment part of the reinforcement learning framework, while the agent includes the three length parameters in the unit cell. In this framework, the agent executes an action to increase or decrease the three length parameters, and calculates the bandgap width before and after the action through environment. Compare the change of bandgap width. If the width increases, it will be rewarded, otherwise it will be punished. During the training, the agent will continuously adjust the three length parameters, and the bandgap width will gradually increase to the maximum. The training tests were repeated many times, while choosing different paths of the parameters evolution and all of them finally converged to the region where the bandgap width is maximized. In addition to this work, we also discussed the topological properties of phononic beams, and realized the structural inverse design with specified topological properties by using TNN (see Figure 8(c)). We discretized the frequency domain according to the different topological properties of the bandgaps and obtained the dataset analytically. Through data training, TNN can map the structures obeyed by the specific topological properties, which allows us to design the topological interface mode at the end.

Another attractive work on mechanical beams was done by Wu et al. [125]. The highlight of this work is that they used neural networks to replace the element concept in the conventional discretization methods for non-periodic system, see Figure 8(d). They train neural network to connect the input and output response of an individual material unit. In addition to the first unit, the other units take the output response of the previous unit as the input response. By simply defining the boundary conditions and frequency requirements, the cross-sectional area of each unit can be adjusted by the minimisation condition between the overall output response and the target output response, thus achieving the overall design.

**Figure 9:**
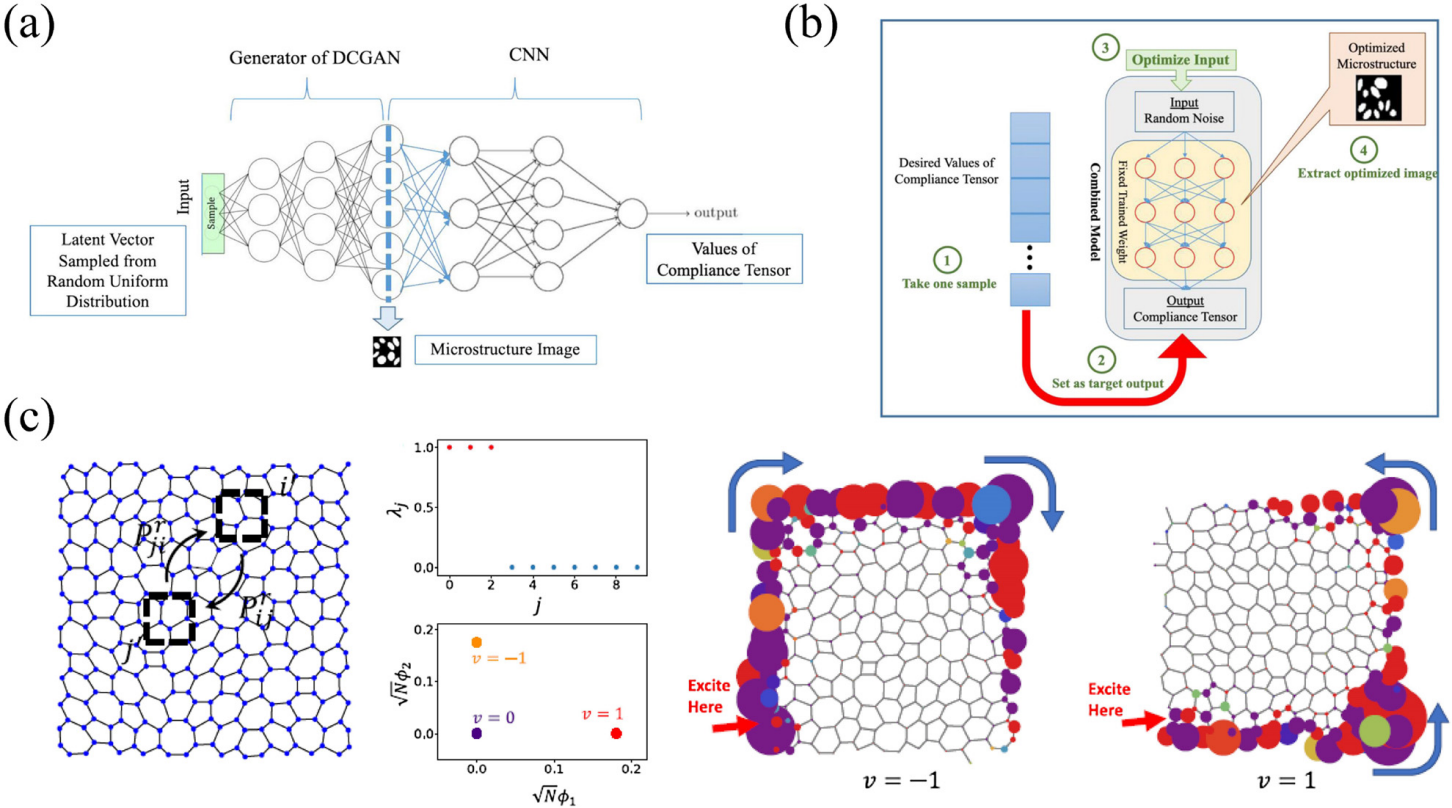
Application cases of generative model and unsupervised manifold clustering for elastic metamaterials. (a) and (b) is the process for the design of 2D porous microstructures [126]. (c) Classification of topological phononic crystals based on unsupervised manifold clustering [133].

We have already discussed the ability of generative models in the section devoted to acoustic metamaterials. The generative models are indispensable direction for future development, so it is natural for researchers to apply them to study elastic metamaterials. Tan et al. reported a deep learning model based on deep convolutional GAN for the design of 2D porous microstructures with specified compliance tensors [126]. Unlike ordinary GAN or CGAN, they connected the tail of the trained GAN generator to a CNN, see Figure 9(a). The generator is responsible for generating the microstructure that meets the geometric constraints according to the latent vector, while the CNN is responsible for obtaining the mechanical properties of the structure. By reasonably defining the cost function using the desired and the predicted compliance tensor, and performing back-propagation on the model, the best latent vector can be found, then the corresponding microstructure can be obtained from the intermediate layer. The flowchart of inverse design process is shown in Figure 9(b). Based on the conventional GAN, this process has designed a variant network structure so that the model can be optimized to obtain a structure with target mechanical properties, which is even more goal-oriented than CGAN. Due to the superiority of GAN and its variant combination models in constructing complex topological structures, some researchers have also used them to design architecture materials [127], lightweight metamaterials [128], and so forth.

Topological invariants which provide an elegant framework for classifying topological properties have different expressions in different spatial dimensions. With the discovery of new materials, topological invariants are becoming more and more important in modern physics. Since topological invariants are an abstract concept, they are often difficult to be properly defined and measured, which has become a major challenge. But actually, the topological properties are embedded in the overall structure feature, so it can be extracted effectively by means of some algorithms. As a powerful tool for solving classification problems, unsupervised manifold clustering algorithm has some basis for identifying topological phase transitions in quantum physics [129], [130], [131], [132]. Inspired by the above works, Long et al. demonstrated a method of topological phononic crystals classification using unsupervised manifold clustering [133]. They use the real space projection operator of the finite phonon lattice to describe the correlation between oscillators, and employed diffusion map method to extract feature and successfully classify phononic crystals according to different topological properties (see Figure 9(c)). On this basis, the approach has been applied to a 1D Su–Schrieffer–Heeger-type phononic chain with random couplings, amorphous phononic topological insulators, higher-order phononic topological states, and a non-Hermitian phononic chain with random dissipations. This work shows that the nonlinear dimensionality reduction ability of unsupervised manifold clustering provides an effective topological phononic crystals classification scheme without *a priori* knowledge of topological families and more especially the need of defining topological invariants.

## 4 ML on atomic-scale phononic metamaterials

2D materials with atomically thick structures have attracted wide attention due to their advantages such as easy preparation, excellent performance, and abundant raw materials. Different from the macro-scale phononic metamaterials introduced in the previous sections, it is a very complex and expensive experimental process to establish the effective relationship between structural information and mechanical properties for atomic-scale metamaterials, although it seems to be just a forward process. Therefore, developing efficient methods to study the mechanical properties of atomic-scale phononic metamaterials is very important to reduce the expensive and time-consuming experimental cost. We now focus on the efforts of machine learning in the study of atomic-scale phononic metamaterials properties in this section.

Thermal conductivity is among the most important properties of a material. For the majority of applications, materials with high thermal conductivities are preferable to enhance the heat transfer and avoid overheating issues. On the other hand, for the thermal insulating and thermoelectric applications, materials with lower thermal conductivities are more desirable in order to reduce the thermal energy loss and improve thermoelectric figure of merit, respectively. By taking a glance on the extensive theoretical studies available in the literature, it can be concluded that the classical molecular dynamics (MD) simulations and density functional theory (DFT) plus Boltzmann transportation equation (BTE) are the currently the most popular methods to predict the thermal conductivity [134], [135], [136]. Modeling of the thermal conductivity by the molecular dynamics simulations are mostly conducted either by the non-equilibrium molecular dynamics (NEMD) or equilibrium molecular dynamics (EMD) methods [137]. The accuracy of MD results however strongly depends on the accuracy of the interatomic potentials that are used to describe the energies and interatomic forces. Moreover, depending on the applied loading and boundary conditions the predictions by MD method may change. In this way, when employing the NEMD method, limited to fixed boundary conditions, the length effect on the thermal conductivity must be elaborately examined. With the EMD technique where periodic boundary conditions are applied the size effect is less problematic, but the formula that is employed to calculate the heat-current may substantially affect the predicted thermal conductivity [138]. These technical issues can justify the large dispersion in the MD-based estimates of the thermal conductivity of 2D materials. Nonetheless, the most critical bottleneck of MD simulations on the basis of empirical interatomic potentials is that for novel materials and structures, an accurate interatomic potential may not exist, which can make the results of the simulations unreliable.

First-principles DFT-based results are well-known to yield highly accurate predictions for the intrinsic properties. Nevertheless, depending on the use of different exchange–correlation functionals combined with ultrasoft or projector augmented wave pseudopotentials, the calculated phonon dispersions may change. When using the DFT–BTE method for the evaluation of the lattice thermal conductivity, the final predictions may change depending on the plane waves cut-off energy, *K*-point mesh size, the size of supercells in the calculation of harmonic and anharmonic force-constants, *Q*-grid size and the cut-off distance in the evaluation of anharmonic force-constants. Various choices for the aforementioned setups of DFT + BTE method may lead to differences in the calculated thermal conductivities, which further highlights the complexity of the problem even when using the first-principles based methods.

To address the thermal transport in 2D materials, machine-learning interatomic potentials (MLIPs) show the greatest potentials. MLIPs are a type of nonparametric designed interatomic potentials with the aim of providing accuracy in the order of quantum mechanics computations, while their computational costs are in the order of empirical interatomic potentials. Neural network potentials (NNP) [139, 140], spectral neighbor analysis potential (SNAP), moment tensor potentials (MTPs) [141] and Gaussian approximation potentials (GAP) [142] are four conventional approaches in creating nonparametric potentials. MLIPs are trained over the DFT datasets, and thus exhibit the same order of accuracy and inherent flexibility to study novel materials. MLIP-based MD calculations can be also conducted using the same platform as that of the common MD calculations on the basis of empirical interatomic potentials.

**Figure 10:**
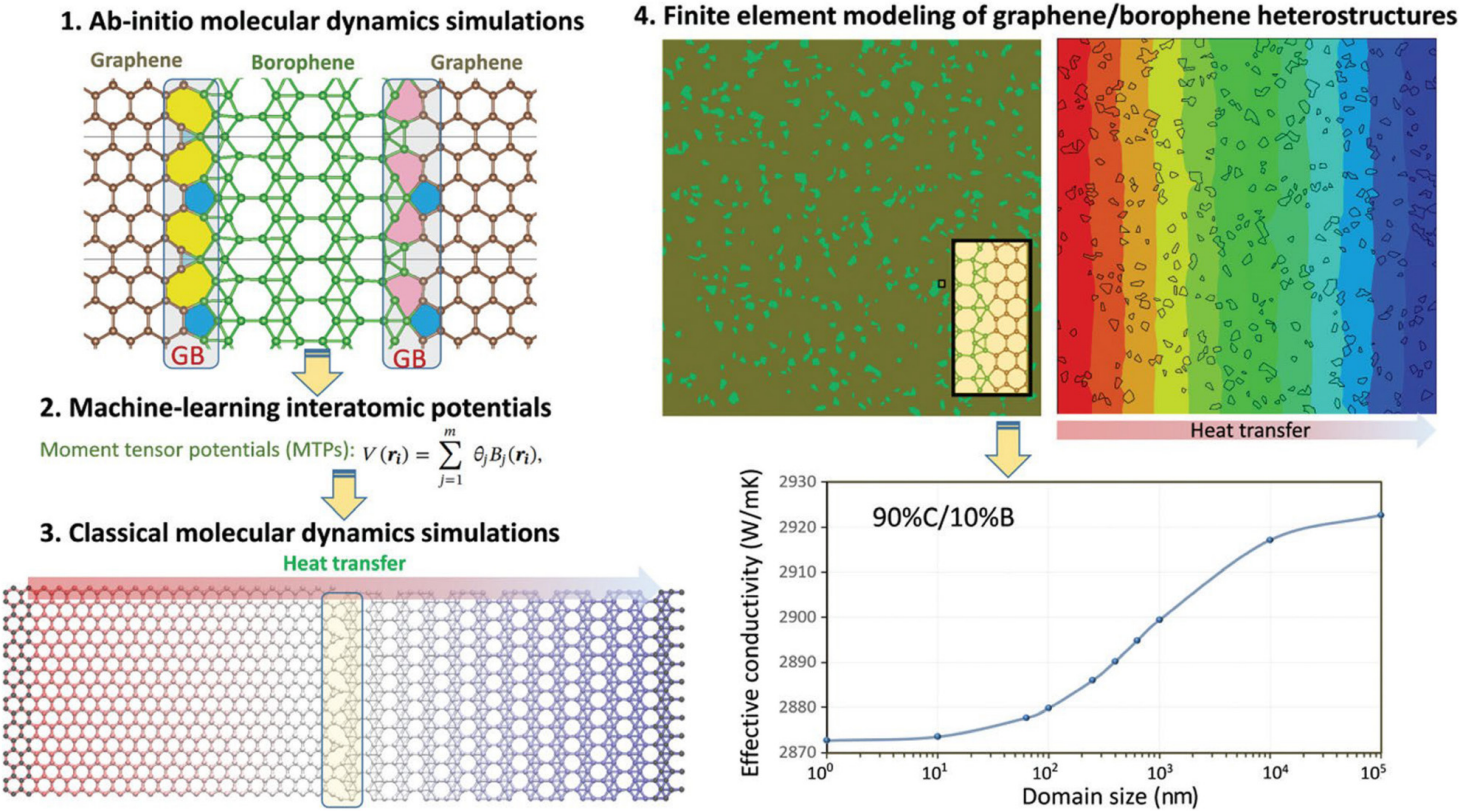
First-principles multiscale modeling of lattice thermal conductivity [148].

In order to briefly explain how MLIPs can address the critical challenges in the evaluation of lattice thermal conductivity of a given material, we would like to categorize materials as those with high and low symmetrical lattices. For highly symmetrical structures like graphene, the BTE solution is more efficient than classical MD simulations. The main computational bottleneck of BTE method is to acquire the anharmonic interatomic force constants. In a recent study for the thermal conductivity of several bulk and 2D structures, it has been confirmed that MLIPs can substantially accelerate the evaluation of anharmonic interatomic force constants while they show close agreement with DFT-based estimations [143]. According to full DFT-BTE based studies [144, 145], it has been suggested that the type of exchange–correlation functional can yield substantial effects on the estimated thermal conductivity of graphene. In a sharp contrast with earlier theoretical studies [144, 145], the estimations by the MLIP-based BTE confirmed the effects of exchange–correlation functional is negligible for the examination of lattice thermal conductivity of graphene [143]. Interestingly, a latest theoretical study [146] confirmed the findings by MLIP-based BTE estimation for the thermal conductivity of graphene and discussed that the scattering in the earlier full-DFT predictions can be associated with not accurately capturing the quadratic dispersion of ZA acoustic branch of graphene. In a recent theoretical work by Liy et al. [146] they extended the earlier work by Mortazavi et al. [143] and included the four phonon scattering in the evaluation of lattice thermal conductivity and they reported excellent agreements between MTP-based and full-DFT estimations for the complex phononic properties. It seems that, MLIPs not only can provide accurate estimations and substantially accelerate calculations, but they can also yield smoother phonon dispersions [147] than computationally demanding DFT calculations and thus reduce the complexity of thermal conductivity examination. Although the BTE solution of lattice thermal conductivity offers a comprehensive and in-depth understanding of phononic heat transport in a material, for low-symmetrical structures it becomes exceedingly computational demanding. Moreover, for the analysis of heat transport of complex structures as those in heterostructures and defective systems, this method becomes computationally infeasible. For these cases, MLIP-based MD simulations can offer unique opportunities, as it has been confirmed in recent studies [148, 149]. One advantage of MLIP-based MD in comparison with BTE solution of thermal conductivity is that with MD simulations multi-phonon scattering can be considered. MLIPs have been also shown to offer the possibility of first-principles multiscale modeling [148, 150], and they can enable a straightforward route to bridge ab-initio level accuracy and flexibility to explore not only the complex heat transport but also the mechanical/failure response of nanostructures at continuum scale [148, 150]. The steps for such a robust possibility are schematically shown in Figure 10, for the analysis of heat transport in graphene/borophene coplanar heterostructures.

## 5 Summary and prospective

In this review, we have introduced the basic principles of the mainstream algorithms of ML and how they are combined with phononic metamaterials. We have highlighted some representative works in this field within the past few years, and the algorithms involved include a series of supervised and unsupervised neural network models, unsupervised manifold clustering, reinforcement learning. These techniques can keenly grasp the laws underlying physical relationships and present them in another form to help us predict properties or inverse design the structures. On the one hand, with the continuous development of acoustics and mechanics, the properties characterization and structural design of metamaterials is not only a key objective of fundamental research, but also a premise for important applications. On the other hand, ML is in a period of vigorous development. Numerous algorithms are constantly innovating and penetrating various disciplines. Meanwhile, advances in computer science provide hardware support. This emerging field of intersection between several disciplines offers researchers a wide range of openings and perspectives, and is expected to have further revolutionary developments in the near future. With the deepening of scientific content and increasing research investment, we anticipate that the next stage will have the following development directions.

– Some high-property implementations, such as strong localization, high robust transmission, low broadband vibration and noise reduction, need intelligent design urgently. The complex relationship between structure and response, such as acoustic-structure interaction in underwater metastructures, also needs the help of data-driven method. Applying ML algorithms to the design of corresponding requirements may take an important step for the practical applications of high-property equipments.
– A series of variant models based on neural network, such as GAN, TNN, etc., whether supervised or unsupervised, can construct the model required by a specific problem after some reasonable combination. We expect future work on these models for the design of acoustic, mechanical and/or hierarchical structures to emerge in abundance.
– Reinforcement learning, as an algorithm developed from behaviorism psychology, is very consistent with the process of human learning and understanding of the world. At present, there are a few reports in the field of acoustic and elastic metamaterial design. It is believed that reinforcement learning, including deep reinforcement learning, will have a trend of further penetration and development in the future.
– Some mature physical mechanisms have similar characteristics to ML algorithms, so it is a new research field to use some internal mechanisms of ML algorithms to understand and realize the simulation of physical mechanisms.
– There is no doubt that MLIP is an effective and accurate tool for atomic-scale modeling and accelerating the evaluation of materials’ properties. However, there are still some challenges in the current methods. Worthy to note that the computational costs of MLIP-based molecular dynamics simulations are by a few orders of magnitude higher than commonly used empirical-based counterparts. In this regard, capturing the Van der Waals and electrostatic long-range interactions are another critical challenge of current MLIP models. The next challenge is the limited transferability of MLIP based models. These bottlenecks of current MLIP models reveal that despite highly promising achievements, there still exists some room for additional extensive research in the field.

Since the development of phononic metamaterials and the demand for various acoustic/mechanical devices will continue to increase, we anticipate that the addition of ML may have very promising prospects.
